# Relationship of sleep duration with incident cardiovascular outcomes: a prospective study of 33,883 adults in a general population

**DOI:** 10.1186/s12889-023-15042-x

**Published:** 2023-01-18

**Authors:** Hui Cui, Rong Xu, Yiming Wan, Yong Ling, Yonggen Jiang, Yiling Wu, Ying Guan, Qi Zhao, Genming Zhao, Maryam Zaid

**Affiliations:** 1 Department of Epidemiology, School of Public Health, Fudan University, Shanghai, 200032 China; 2 Songjiang District Center for Disease Control and Prevention, Shanghai, 200032 China; 3 Department of Social Medicine, School of Public Health, Fudan University, Shanghai, 200032 China

**Keywords:** Sleep duration, Cardiovascular disease, Cohort studies, Epidemiology

## Abstract

**Background:**

Studies on the effect of sleep duration on cardiovascular health have contradictory findings. Underlying health issues may have led to inconsistent results and warrant consideration. We aim to assess the relationship of night sleep duration with incident cardiovascular disease (CVD) in a general population, taking into consideration underlying chronic diseases.

**Methods:**

Data from Shanghai Suburban Adult Cohort and Biobank with a median follow-up of 5.1 years was used, including 33,883 adults aged 20–74 years old. Incident CVD cases were reported and recorded by the Center for Disease Prevention and Control in Songjiang, Shanghai. We used Cox proportional hazard regression models and restricted cubic spline (RCS) analysis to explore the relationship between different sleep groups and sleep duration with incident CVD outcomes, through stratification by gender and age, as well as different health conditions, with adjustments for potential confounders.

**Results:**

Long sleep duration (> 9 h) compared to > 7 to ≤ 8 h was associated with overall incident CVD in participants aged ≥ 50 years old: HR(95%CI) = 2.07 (1.15, 3.74) for 50-59y and 1.43 (1.04, 1.93) for 60-74y. RCS analysis showed a J-shaped relationship between sleep and CVD risk in those ≥ 50y, which was confirmed only in those with a chronic health condition. Non-linear relationships between sleep and CVD risk factors, such as BMI, blood glucose and glycated haemoglobin, were observed.

**Conclusions:**

Long sleep duration is associated with increased risk of CVD in people ≥ 50y. However, CVD risk factors and underlying health conditions such as hypertension, and diabetes, may play a driving role in the relationship.

**Supplementary Information:**

The online version contains supplementary material available at 10.1186/s12889-023-15042-x.

## Background

Cardiovascular diseases (CVD) are the leading cause of death worldwide and over 80% are due to heart attacks and strokes [1]. However, one-third of these deaths occur prematurely in people aged under 70 years old, which means that CVD is preventable in the population and that it is important to explore the potential risk factors. As a modifiable lifestyle, sleep is closely associated with cardiovascular functions, such as heart rate, blood pressure, or cardiac output [2]. Changes in society and technology influence sleep in many aspects, including sleep quality and sleep duration in the population [3, 4]. A study on sleep trends among adults in the United States showed that the probability of short sleep in the population gradually increased from 2007 to 2017 [5]. Also, it was reported that overall reductions in Chinese daily average sleep duration were observed from 2010 to 2018 [6].

Although there were some studies on the association between sleep duration and risk of CVD incidence, contrasting results were found [7–10]. The Morgen study [7] carried out in the Netherlands between 1993 and 1997 supported that short sleep duration was associated with predicted 12-year CVD incidence, whereas long sleep duration was not. Results of the Jichi Medical School Cohort Study [8] from Japan indicated that only in male participants whose sleep duration was shorter than 6 h, the incidence risk of CVD events was higher than in those who slept 7 to 7.9 h per night. In another prospective study in 2015 from the MONICA-Brianza and PAMELA population-based cohorts [9], a significant increase in hazard ratio of CVD events was observed for participants who slept longer than 9 h with respect to 7–8 h per night. However, a study utilizing UK Biobank cohort data [10] found that short (≤ 5 h) and long (≥ 9 h) sleep duration were both associated with increased risk of CVD incidence and mortality, even after multivariate adjustment. More recently, a prospective cohort study from the US indicated that individuals who slept around 7 h per night had the lowest CVD-specific mortality compared with those who slept shorter than 6 h or longer than 8 h [11]. Nevertheless, there is a lack of understanding of how sleep duration is related to CVD incidence risk in a community-based general population. Major confounders, such as age and the presence of other health conditions, such as diabetes, hypertension and obesity, may have influenced the findings and led to contrasting results. The risk of CVD might vary in sleep duration in populations with different health statuses. Therefore, further studies are required to focus on the role of health conditions in the association between sleep duration and CVD outcomes. Moreover, research so far is limited on the relationship of conventional risk factors with sleep duration, which is valuable for exploring potential mechanisms between sleep duration and CVD incidence. Modern cohorts, in which there is likely a broader range of sleep duration due to the influence of a modern lifestyle, need to be investigated. Thus, we aim to assess the longitudinal relationship between sleep duration and incident CVD as well as describe the potential metabolic relationship by careful assessment of health conditions and conventional CVD risk factors in a modern population-based cohort.

## Methods

### Study population

From April 6, 2016 to October 31, 2017, the Shanghai Suburban Adult Cohort and Biobank (SSACB) recruited 37,670 residents (aged 20 to 74 years old) from the general population living in Songjiang District, Shanghai, China. The study protocol and details have been published elsewhere [12]. In brief, the participation rate was 90% and participants attended a local community health centre, where they were invited to take physical examinations, answer questionnaires, and provide blood and urine samples. Written informed consent from each participant was obtained before enrollment. A flow chart of study population enrollment can be seen in Additional file 1. Participants were excluded from analysis if they had duplicated ID numbers (*n* = 2), or missing information on sleep duration (*n* = 96), or history of CVD (*n* = 2423). After these exclusions, a total of 33,883 eligible adults were included in the present analysis.

### Questionnaire and anthropometric measurements

Data were collected using questionnaires on demographics (age, gender, education level, marital status, and occupation), lifestyle factors (tobacco smoking, alcohol drinking, and physical activity), disease history (cardiovascular and cerebrovascular diseases, depression, and other diseases), and menopausal status in females. Education level was categorized into four groups: “primary school”, “middle school”, “high school” and “college and above”. Occupation was grouped as “officer”, “Businessman/Servicer”, “Manual worker”, or “others/retired”. Smoking was defined as having smoked at least one cigarette per day for more than 6 months. Alcohol intake was defined as drinking at least three times per week for more than 6 months. Information on physical activity in the past year was acquired from each participant and based on metabolic equivalent tasks (METs) multiplied by the total minutes per week (METs-min/week) [13], it was divided into tertiles (low: < 2880; moderate: 2880–5040; and high: ≥ 5040). Trained investigators measured height and weight, then body mass index (BMI) was computed as weight (in kilograms) divided by height (in meters) squared. Obesity was defined as BMI ≥ 28.0 kg/m^2^according to the National Health and Family Commission in China [14]. Blood pressure was averaged from three measurements taken after participants rested for 5 min. Hypertension was defined as self-reported hypertension during the face-to-face interview, or abnormal blood pressure tested in the physical examination, including systolic blood pressure (SBP) ≥ 140 mmHg and/or diastolic blood pressure (DBP) ≥ 90 mmHg. Diabetes was defined as self-reported diabetes in the questionnaire, or fasting plasma glucose (FPG) ≥ 7.0 mmol/L in blood testing. Chinese guidelines [15] were used for the diagnosis of dyslipidemia, which was defined as either low-density lipoprotein cholesterol (LDL-C) ≥ 4.1 mmol/L, total cholesterol (TC) ≥ 6.2 mmol/L, fasting triglycerides (TG) ≥ 2.3 mmol/L, high-density lipoprotein cholesterol (HDL-C) < 1.0 mmol/L or self-reported hyperlipidemia. Marital status was divided into married, divorced, widow (or widower), and unmarried. The use of sleep medication was classified into four categories: none, < 1 time/week, 1 ~ 2 times/week, and ≥ 3 times/week. Menopause status of females was separated into premenopausal, natural menopause, and induced menopause. Information on chronic disease history was also collected through questionnaires (self-report), including hypertension, diabetes and dyslipidemia.

### Sleep duration

Participants were asked (either in Mandarin or in Shanghainese) “During the past month, when did you usually go to bed at night?” and “During the past month, when did you usually get up in the morning?” Each answer contained hours and minutes. The sleep duration of each participant was calculated as the time difference between these two answers. The range of sleep duration of all participants was between 3 to 16 h. Four mutually exclusive sleep duration groups were created as “ ≤ 7 h”, “ > 7 h to ≤ 8 h”, “ > 8 h to ≤ 9 h” and “ > 9 h” respectively, in which the “ > 7 h to ≤ 8 h” was set as the reference group.

### Main outcomes

The following codes from the International Statistical Classification of Diseases and Related Health Problems, Tenth Revision (ICD-10) were used to define outcomes: CVD (I21, I22, I60, I61, I62, I63, I63.8, I64) and CVD death (I25.9, I46.1). In detail, the outcomes were incident cerebral infarction (non-lacunar cerebral infarction or lacunar cerebral infarction), intracranial haemorrhage (intra-parenchymal haemorrhage or non-intra-parenchymal haemorrhage), subarachnoid haemorrhage, stroke (not specified), and acute myocardial infarction, and CVD death resulting from coronary heart disease or sudden cardiac death. Vital statuses and incident dates were provided by Shanghai Songjiang Center for Disease Control and were matched with participants of this study. Censor was defined as a participant who lost to follow-up, died from non-CVD diseases, or reached the endpoint of the study (February 28, 2022). Follow-up time was counted for each participant from the date of baseline investigation to the date of the first CVD event or study end date, whichever occurred first. In this study, incident CVD events were defined as participants who were diagnosed with at least one of the above outcomes during follow-up.

### Statistical analysis

The characteristics of participants were described within four groups of sleep duration. Mean (standard deviation, SD) was presented for normally distributed variables, median (interquartile range) was presented for skewed variables, and frequency percentage was presented for categorical variables. Moreover, the statistical differences in characteristics between the four groups were analyzed by ANOVA (for means), Kruskal–Wallis (for medians), and Chi-square analysis (for frequencies).

We performed age-stratified analysis by dividing all participants into three groups: 20-49y, 50-59y, and 60-74y. COX proportional hazards models were used to analyze the associations of sleep duration with incident CVD. Model 1 was adjusted for age, gender, marital status, education level, and occupation and Model 2 was further adjusted for smoking, alcohol drinking, physical activity, hypertension, diabetes, dyslipidemia, BMI, sleep medication, and depression. We also assessed the interactions of gender and age, separately, on associations of sleep duration with incident CVD. Risk of CVD incidence among the four sleep duration groups was assessed in participants with or without the following health conditions: hypertension, diabetes, dyslipidemia, and obesity.

Restricted cubic splines with Cox proportional hazard regression models were used to assess the dose–response relationship between sleep duration and incident CVD events. We used 4 knots placed at the 10th, 30th, 60th, and 90th percentiles of sleep duration with 8 h as a reference. The relationship between sleep duration and traditional CVD risk factors, such as BMI and blood pressure, was analyzed with restricted cubic splines regression. SAS version 9.4 was used for all statistical analyses.

## Results

### Baseline characteristics

Table 1 shows the baseline characteristics of study participants. Participants who slept more than 9 h had an average age of 58.2 (SD: 12.0), which is the maximum among the four sleep duration groups. Compared with the other groups, individuals with sleep duration > 9 h were more likely to have hypertension and dyslipidemia, and have higher levels of LDL-C, triglycerides, and total cholesterol at baseline. Moreover, these participants were less likely to be married and had lower proportion of having education at the level of college or above. Participants who slept > 7 to ≤ 8 h per night had the lowest TG level and were the least likely to have diabetes. The lowest median FPG and mean haemoglobin A1C (HbA1C) levels were seen in participants who slept > 8 to ≤ 9 h.

**Table 1:** Baseline characteristics of participants according to groups of sleep duration (*N* = 33,883)

	**Sleep duration (h)**				***P* value**
	** ≤ 7** **(*n* = 6762)**	** > 7 to ≤ 8** **(*n* = 12,369)**	** > 8 to ≤ 9** **(*n* = 10,214)**	** > 9** **(*n* = 4538)**	
**Age (y)**	55.7 (10.2)	55.0 (11.1)	55.8 (11.7)	58.2 (12.0)	< 0.001
**Male (%)**	45.3	38.1	38.4	43.8	< 0.001
**SBP (mmHg)**	133.0 (19.2)	133.2 (19.3)	133.3 (19.6)	138.8 (19.4)	< 0.001
**DBP (mmHg)**	80.0 (10.8)	79.9 (10.6)	80.0 (10.6)	80.5 (10.2)	0.018
**BMI (kg/m**^**2**^**)**	24.7 (3.3)	24.3 (3.3)	24.2 (3.3)	24.3 (3.4)	< 0.001
**HDL-C (mmol/L)**	1.40 (0.34)	1.42 (0.34)	1.42 (0.36)	1.41 (0.41)	0.004
**LDL-C (mmol/L)**	2.76 (0.83)	2.78 (0.83)	2.79 (0.83)	2.81 (0.84)	0.016
**TG (mmol/L)**	1.35 (0.98,1.92)	1.33 (0.96,1.89)	1.34 (0.97,1.91)	1.37 (0.99,1.97)	0.001
**TC (mmol/L)**	4.92 (0.93)	4.93 (0.93)	4.95 (0.94)	4.98 (0.94)	0.008
**FPG (mmol/L)**	4.75 (4.30,5.38)	4.72 (4.26,5.35)	4.70 (4.25,5.35)	4.75 (4.25,5.48)	< 0.001
**HbA1C (%)**	5.82 (0.85)	5.76 (0.82)	5.74 (0.84)	5.79 (0.94)	< 0.001
**Physical activity (%)**					< 0.001
low	34.4	33.7	36.0	37.2	
moderate	30.8	31.2	31.2	32.6	
high	34.8	35.1	32.9	30.3	
**Smoking (% yes)**	29.4	21.8	21.5	24.8	< 0.001
**Alcohol intake (% yes)**	16.1	12.6	12.1	16.2	< 0.001
**Hypertension (% yes)**	50.0	49.0	50.2	55.2	< 0.001
**Diabetes (% yes)**	10.5	9.2	9.9	12.1	< 0.001
**Dyslipidemia (% yes)**	54.7	53.6	54.9	56.9	0.002
**Obesity (% yes)**	16.3	14.0	13.7	14.3	< 0.001
**Depression (% yes)**	0.5	0.4	0.5	0.5	0.594
**Sleep medication (%)**					0.003
Never	97.4	97.6	97.6	97.1	
< 1/week	1.1	0.9	0.8	0.8	
1 ~ 2/week	0.7	0.6	0.8	0.7	
≥ 3/week	0.8	0.9	0.9	1.5	
**Marital status (%)**					< 0.001
Married	92.9	93.4	93.4	91.4	
Divorced	1.7	1.4	1.1	1.1	
Widow/widower	4.3	3.8	4.1	5.4	
Unmarried	1.1	1.3	1.3	2.1	
**Education (%)**					< 0.001
Junior middle or lower	41.9	41.2	47.4	57.9	
Senior middle school	49.7	48.4	42.9	34.3	
College or above	8.4	10.4	9.7	7.7	
**Occupation (%)**					< 0.001
Officer	37.4	36.3	32.4	25.2	
Businessman/servicer	14.9	14	14.8	13.4	
Manual worker	24.7	28.1	32.8	38.2	
Others/retired	23.0	21.7	20.0	23.2	

Data are presented as mean (SD) for normally distributed variables, median (interquartile range) for skewed variables, or frequency percentage for categorical variables

*P* values were obtained from ANOVA, Wilcoxon, or Chi-square analysis (respectively) across sleep duration groups

*SBP* Systolic blood pressure, *DBP* Diastolic blood pressure, *BMI* Body mass index, *HDL-C* High-density lipoprotein cholesterol, *LDL-C* Low-density lipoprotein cholesterol, *TG* Triglycerides, *TC* Total cholesterol, *FPG* Fasting plasma glucose, *HbA1C* Haemoglobin A1C

### Associations of sleep duration with incident CVD

During a median follow-up of 5.1 years, 540 participants experienced CVD events, of which 512 had incident CVD, and 28 died of CVD. Longer sleep duration of more than 9 h was independently associated with a higher risk of CVD incidence, both in males and females, even after adjustments for potential confounders [see Additional file 2]. No gender interaction was present in the relationship between sleep duration and incident CVD, thus male and female data were combined in further analyses.

Table 2 shows the risk HR (95% CI) of incident CVD in the four sleep duration groups across age strata. Individuals aged 20-49y in the > 9 h group had HR of 1.37 (0.63, 5.71) for incident CVD (Model 2). For subjects aged 50-59y, the HR (95% CI) of incident CVD in > 9 h was nearly double that of the reference > 7 to ≤ 8 h group; Model 2 HR (95% CI) of 2.07 (1.15, 3.74). Similarly, for those aged 60-74y, the risk of incident CVD with longer sleep duration was 43% higher (Model 2 HR = 1.43; 95%CI, 1.04, 1.93) than that of the reference group. Detailed age-specific analysis shows a similar result [see Additional file 3] Cumulative incidence curves of all participants and those aged ≥ 50y show a higher cumulative incidence of CVD in participants with ≥ 9 sleep hours compared to other groups [see Additional file 4].

**Table 2:** Hazard ratios (95% CI) of incident CVD according to sleep duration and age groups in all participants (*N* = 33,883)

Age Group and Model	Sleep duration (h)			
	** ≤ 7**	** > 7 to ≤ 8**	** > 8 to ≤ 9**	** > 9**
**20-49y (*N* = 8039)**				
CVD Incidence (N)	7	9	5	5
Model 1	1.41 (0.52, 3.79)	1 (REF)	0.72 (0.24, 2.15)	1.90 (0.63, 5.71)
Model 2	1.29 (0.44, 3.75)	1 (REF)	0.77 (0.25, 2.37)	1.37(0.36, 5.21)
**50-59y (*N* = 11,383)**				
CVD Incidence (N)	29	39	39	20
Model 1	1.13 (0.70, 1.83)	1 (REF)	1.38 (0.88, 2.15)	1.96 (1.14, 3.37) ^*^
Model 2	1.27 (0.76, 2.12)	1 (REF)	1.52 (0.95, 2.45)	2.07 (1.15, 3.74) ^*^
**60-74y (*N* = 14,461)**				
CVD Incidence (N)	68	110	117	92
Model 1	1.13 (0.84, 1.53)	1 (REF)	1.11 (0.86, 1.45)	1.47 (1.12, 1.95) ^*^
Model 2	1.18 (0.86, 1.62)	1 (REF)	1.06 (0.80, 1.40)	1.43 (1.04, 1.93) ^*^

Model 1 is adjusted for age, gender, marital status, education level, and occupation; Model 2 is adjusted for Model 1 and further adjusted for smoking, drinking, physical activity, hypertension, diabetes, dyslipidemia, BMI, sleep medication, and depression

*CVD* Cardiovascular disease, *REF* Reference

^*^ denotes statistical significance at the *P* < 0.05 level

**Fig. 1:**
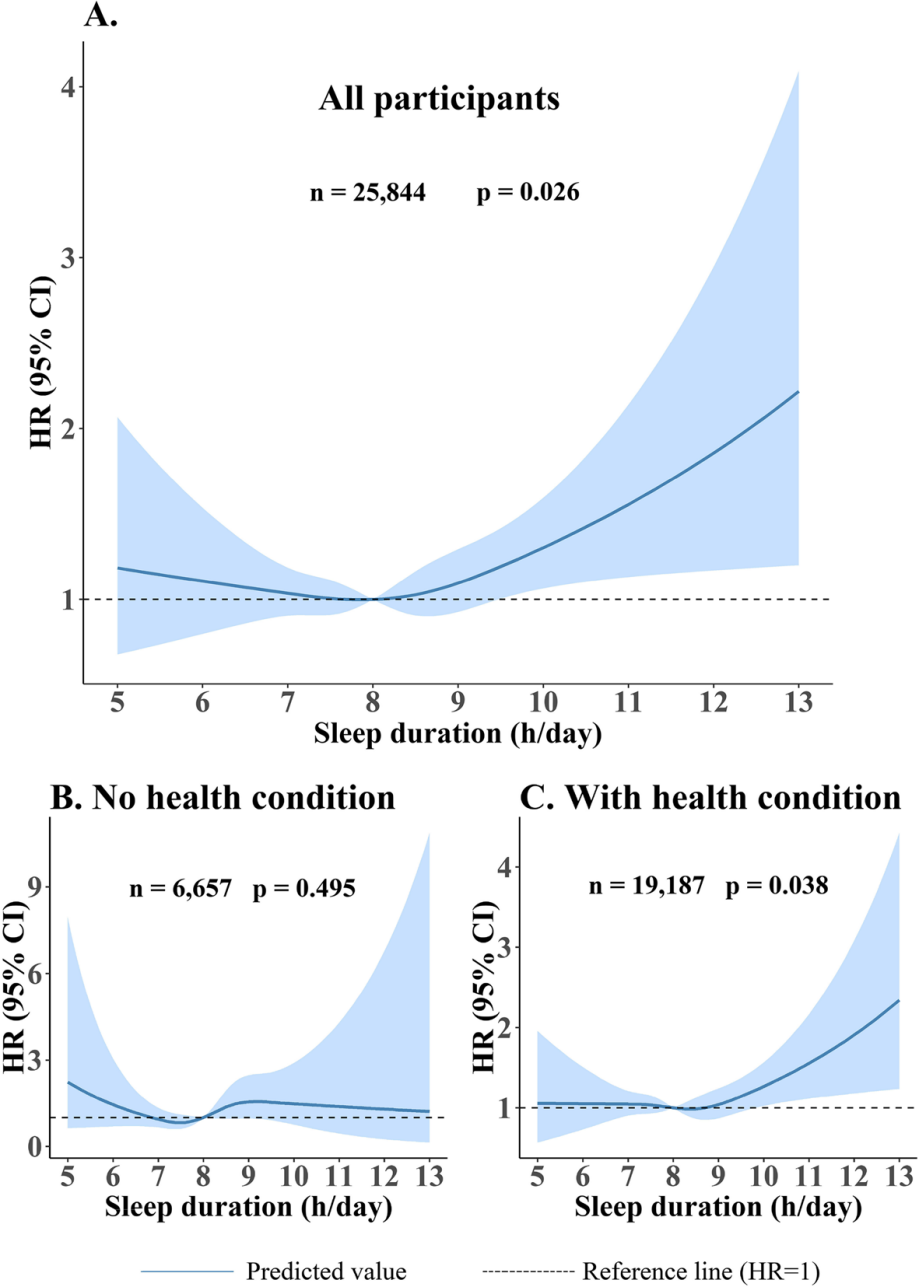
Cubic spline curves for the relationship between sleep duration and incident CVD in participants ≥ 50y (**A**: all participants, **B**: participants without any health conditions, **C**: participants with one or more health conditions). The model is adjusted for age, gender, marital status, education level, occupation, smoking, drinking, physical activity, BMI, sleep medication, and depression. HR: hazard ratio. 8 h/day is used as the reference

Cubic spline analysis revealed a non-linear J-shaped relationship of sleep duration with incident CVD in participants. Figure 1 illustrated a J-shaped relationship between continuous sleep duration and incident CVD risk in participants aged ≥ 50y. Compared to participants with a sleep duration of 8 h (reference), those who slept more than 9 h had a higher risk of incident CVD. After stratification of these participants into those with and without a health condition (such as hypertension, diabetes, dyslipidemia or obesity), the J-shaped relationship was only evident in those with presence of one or more health conditions. In sensitivity analyses with 7 h and 9 h as the reference group respectively, similar results were observed [see Additional file 5].

**Fig. 2:**
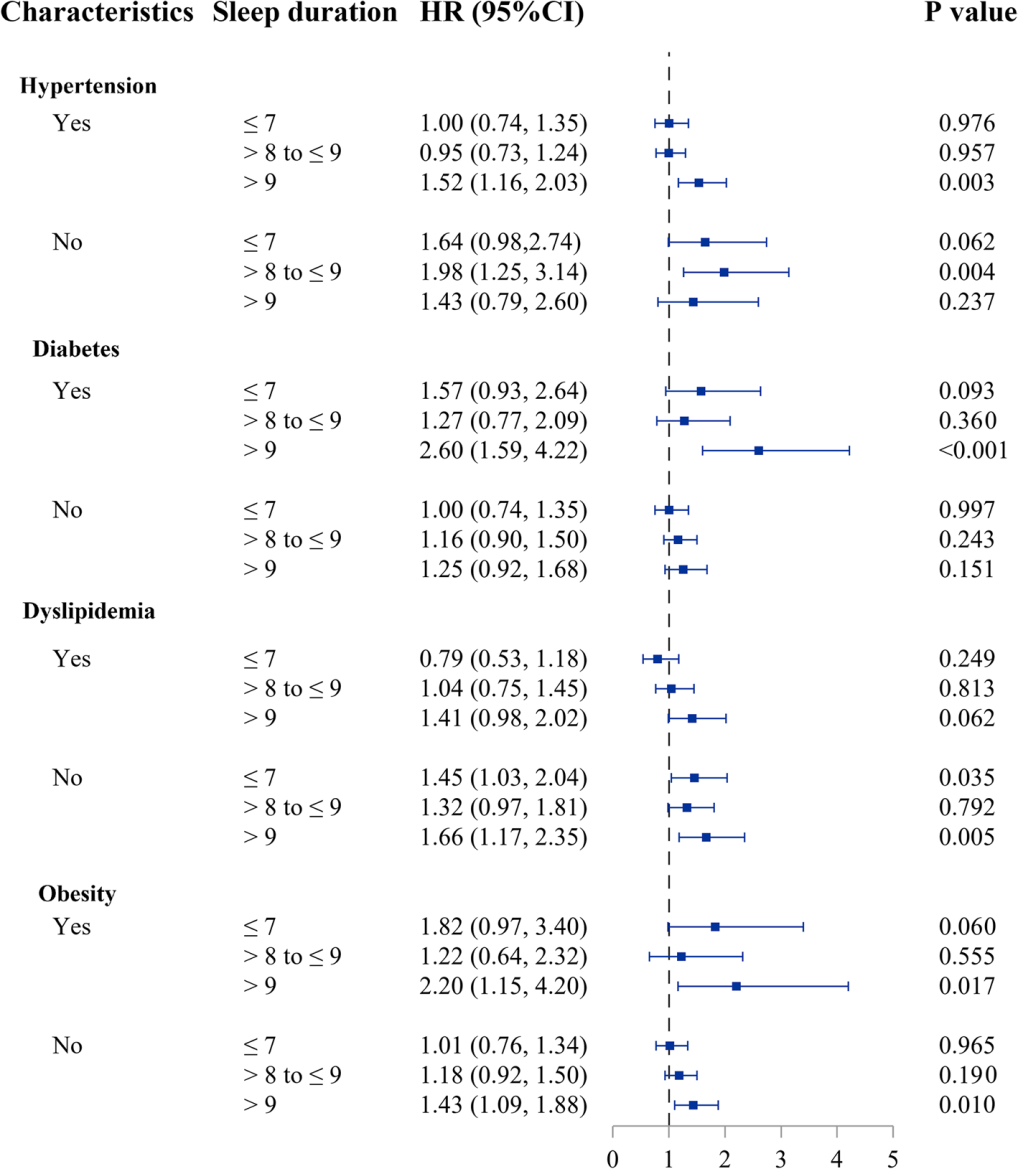
Forest plot of HR (95% CI) between sleep duration and incident CVD in participants ≥ 50y by subgroups of hypertension, diabetes, dyslipidemia, and obesity. The model is adjusted for age, gender, marital status, education level, occupation, smoking, drinking, physical activity, hypertension, diabetes, dyslipidemia, obesity, sleep medication, and depression. > 7 h to ≤ 8 h sleep duration is used as the reference. Each subgroup analysis was adjusted for all the covariates listed above except itself. HR: hazard ratio. CI: confidence interval

To further elucidate which health condition in those aged ≥ 50y influences the relationship between sleep and CVD, we further stratified older participants into with or without certain health conditions: hypertension, diabetes, dyslipidemia, and obesity. Participants with hypertension, diabetes or obesity have a higher risk of CVD in sleep duration > 9 h group compared to the counterpart > 7 h to ≤ 8 h group (Fig. 2). In participants with dyslipidemia, those in the > 9 h group had an HR of 1.41 (0.98, 2.02), although not statistically significant (*p* = 0.064). Furthermore, in participants without dyslipidemia or obesity, those who slept longer than 9 h are at an increased risk of incident CVD compared with that in the reference group. Generally, participants without the said health conditions have higher HR estimates with sleep > 9 h compared to > 7 h to ≤ 8 h, although most were not significant.

**Fig. 3:**
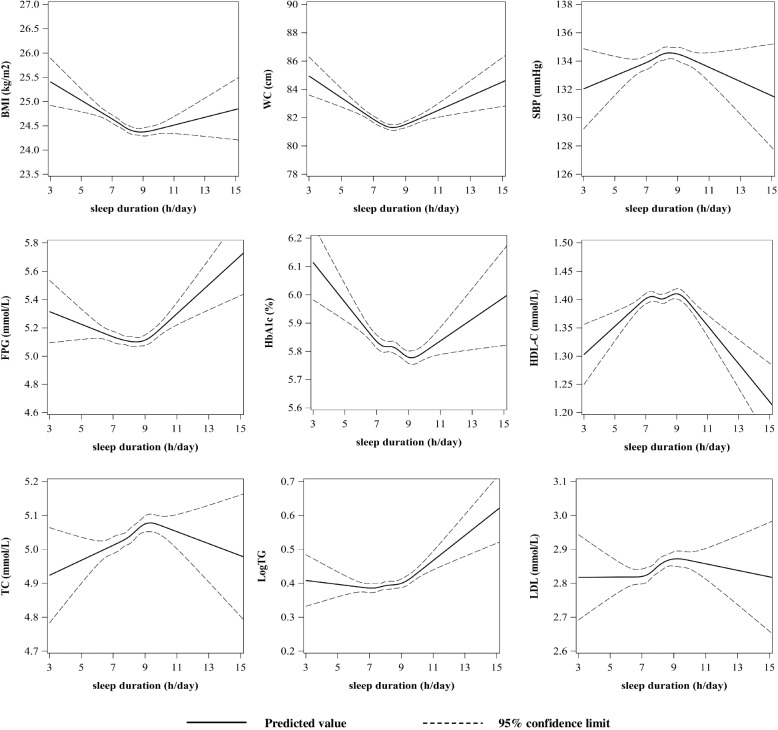
Age-adjusted association of each CVD risk factor with sleep duration. BMI: body mass index; WC: waist circumstance; SBP: systolic blood pressure; FPG: fasting plasma glucose; HbA1C: haemoglobin A1c; HDL-C: high-density lipoprotein cholesterol; TC: total cholesterol; Log TG: Log of triglycerides; LDL-C: low-density lipoprotein cholesterol

Additionally, we examined the relationship between continuous sleep duration and traditional risk factors of CVD in Fig. 3, including physical measurement indicators (BMI, SBP, and waist circumference (WC)), as well as laboratory testing indicators (FPG, HbA1C, TG, TC, LDL-C, and HDL-C). In restricted cubic spline analysis of the age-adjusted association of each CVD risk factor with sleep duration, U-shaped relationships were observed between sleep and BMI, WC, FPG, HbA1C, and log TG. In contrast, a reverse U-shaped relationship was observed between sleep and HDL-C. No distinct relationships were observed between SBP, TC, and LDL-C with sleep.

## Discussion

In the present study, long sleep duration (> 9 h) was observed to be independently associated with an increased risk of incident CVD in participants aged 50y or above, even after adjustment for potential confounders. Recently, an umbrella review of sleep duration with health outcomes [16], including 85 meta-analyses, revealed long sleep was highly associated with increased risk of incidence, development or death of CVD. Although other studies have found that higher risk of CVD is evident under conditions of short sleep duration [10, 17], we have not identified such a relationship in our study. This may have been due to the small number of subjects who reported sleeping less than 5 h and fewer incident cases in these participants, and a relatively short median follow-up period of 5.1 years, all of which may have led to limited statistical power.

Previous studies have shown that age plays a critical role in the relationship between sleep duration and CVD events but the findings were not consistent [9, 17–19]. A meta-analysis consisting of 19 studies [15] found no significant differences between age in the association of sleep duration with cardiovascular outcomes. Nevertheless, in a large cohort of Italian adult men [9], the increased risk of CVD events in long sleepers was only significant for older adults aged above 60y. Another study, Dongfeng-Tongji cohort, including 19,370 Chinese residents conducted a two-level age stratification analysis [18]. As a result, the significant association between long sleep duration and coronary heart disease only was shown in participants aged less than 65 years old, instead of those who were 65y or above. More recently, in a study of participants from 21 countries [19], the J-shaped relationship between sleep duration and major CVD events was consistently observed in participants both under or above 50 years old. These inconsistent conclusions might be driven by different study populations. Based on the wide age range of participants in our study, from 20 to 74 years, we performed age-stratified analysis. Consequently, the higher risk of CVD associated with long sleep duration mainly occurred in participants who were ≥ 50 years old instead of those who were 20-49y. Thus, in general, we found no associations in adults younger than 50 years old. More large population-based prospective studies with a wide age range are needed to confirm the influence of age on the association between sleep duration and CVD outcomes.

With increasing age, there is an increase in average blood pressure, blood glucose, and triglycerides, which may lead to hypertension and diabetes, among other comorbidities and diseases [20]. Thus, to elucidate whether the health status of participants affects the association, we stratified participants ≥ 50 years into “No health condition” or “With health condition” groups, based on absence or presence, respectively, of current chronic diseases. For participants with one or more health conditions, with an increase of sleep duration greater than 8 h, there is a significant rise in CVD incidence risk. However, in participants without any health condition, the non-linear relationship is not exactly consistent with all participants aged above 50y. This demonstrates that health conditions might potentially influence the relationship between long sleep duration and CVD risk. Long sleep duration may reflect the preclinical unhealthy status of participants [21, 22], thus, the relationship between sleep and CVD may be confounded by health disorders, such as hypertension, diabetes, dyslipidemia and obesity. In our stratification analysis of these chronic diseases, a trend of increased CVD risk with longer sleep duration exists in participants with hypertension, diabetes, or obesity. This was also seen in those with dyslipidemia, although not significant (*p* = 0.064). In participants without dyslipidemia or obesity, a similar trend was observed. Sleep duration longer than 9 h was associated with a higher HR estimate of incident CVD. This suggests that sleep itself even in generally healthy individuals may slightly exacerbate risk of CVD. Future work will involve the detailed assessment of possible mediating factors, such as different health conditions, using non-linear functions for survival data.

The biological mechanisms that link long sleep duration with CVD are not clear, although short sleep duration has been widely considered an unhealthy lifestyle for many years [23–25]. Some studies have investigated the mechanism of sleep deprivation with CVD. Abnormal fluctuation of ghrelin and leptin levels, serum lipid levels, and a stressed autonomic nervous system are the main explanations linked to insufficient sleep [26–28]. Regarding long sleep hours, there is increasing evidence for its relationship with increased arterial stiffness, which is a predictor of CVD [29–31]. Moreover, blood pressure variability and diabetes are reckoned to be mediators of the association between long sleep and CVD incidence [32]. To better understand the dose–response relationship between sleep duration and typical markers of metabolic syndrome, which may affect CVD risk, we performed restricted cubic spline analysis adjusted for age. In our results, U-shaped associations of sleep duration with BMI, WC, FPG, HbA1C, and log TG were illustrated, while a reverse U-shaped relationship between sleep and HDL-C was identified. All of these factors have been often associated with increased harm to the cardiovascular system, and manifest through obesity or diabetes [33]. SBP, LDL-C, and TC did not illustrate U-shaped relationships with sleep which may have been influenced by medication for hypertension or dyslipidemia. More experimental studies are needed to explore the biomechanisms between sleep and CVD.

To the best of our knowledge, this is the first study in a general population to carefully assess how different health conditions may affect the association between long sleep duration and CVD incidence. We conducted a longitudinal study in a population-based cohort, which provides an adequate platform for the analysis of exposures and outcomes. With a large sample from a general population, we have a broad age range for analyzing the associations between sleep and CVD through age stratification, which can uncover at-risk groups and reduce possible confounding by age. Moreover, we adjusted for many potential confounders, including demographic and lifestyle factors. Finally, as a rapid urbanized community, the study results from Songjiang district of Shanghai can be meaningful to other cities in China with similar characteristics. However, there are some limitations to our study. First, as we used self-reported sleep of participants at the time of questionnaire survey at baseline, the change in sleep patterns during follow-up could not be assessed. The definition of sleep duration as the difference between the time to go to bed and the time to get up may lead to an overestimation, hence sleep length in the analysis is likely to be higher than the true value. Second, due to a lack of data on midday napping, we were unable to examine the entire duration of sleep in one day. Third, with limited follow-up duration of about 5 years, the number of incident cases was not sufficient to detect a small difference in some stratified analyses, such as the younger age groups.

## Conclusions

Long sleep duration was observed to be associated with risk of CVD in a Chinese suburban population, especially for older adults aged ≥ 50 years. Health conditions such as hypertension, diabetes and obesity, and CVD risk factors, such as blood glucose, HbA1C, BMI and TG, may play a driving role in the relationship. Further epidemiology and laboratory studies are needed to confirm the relationship between sleep duration and CVD in populations with different health conditions, as well as the potential mechanism between sleep and incident CVD.

## Supplementary Information

**Additional file 1.** A flow chart of the study participants included in the present study who were originally recruited from Songjiang District, Shanghai, China as part of the Shanghai Suburban Adult Cohort and Biobank (SSACB). * Inconsistent number is due to overlapped number of participants between data sources.

**Additional file 2:** **Supplementary Table 1.** Hazard ratios (95% CI) of incident CVD in male and female participants by sleep duration.

**Additional file 3:** **Supplementary Table 2.** Hazard ratios (95% CI) of incident CVD according to sleep duration and age groups in all participants (*N* = 33,883).

**Additional file 4.** Cumulative incidence curve in all participants (a) and participants ≥50y (b). Sleephg: sleep hour group. 1: "≤7 h"; 2: ">7 h to ≤ 8h"; 3: ">8 h to ≤9 h"; 4: ">9". CIF = cumulative incidence function.

**Additional file 5.** Spine curves for the relationship between sleep duration and incident CVD in participants aged ≥50y with 7h as the reference (a) and 9h as the reference (b). Upper and lower 95% CI are displayed as dashed lines. The model is adjusted for age, gender, martial status, education level, smoking, drinking, physical activity, hypertension, diabetes, dyslipidemia, BMI, sleep medication, health status, and depression.

## Data Availability

The data that support the findings of this study are available from Maryam Zaid but restrictions apply to the availability of these data, which were used under license for the current study, and so are not publicly available. Data are however available from the authors upon reasonable request and with permission of Maryam Zaid.

## Abbreviations

CVD: Cardiovascular disease
RCS: Restricted cubic spline
SSACB: Shanghai Suburban Adult Cohort and Biobank
BMI: Body mass index
SBP: Systolic blood pressure
DBP: Diastolic blood pressure
FPG: Fasting plasma glucose
LDL-C: Low-density lipoprotein cholesterol
TC: Total cholesterol
TG: Triglycerides
HDL-C: High-density lipoprotein cholesterol
ICD-10: International Statistical Classification of Diseases and Related Health Problems, Tenth Revision
SD: Standard deviation
WC: Waist circumference
HbA1C: Haemoglobin A1C
REF: Reference
CI: Confidence interval
