# Supplementary material for: Relationship of sleep duration with incident cardiovascular outcomes: a prospective study of 33,883 adults in a general population
Source: BMC Public Health. 2023 Jan 18;23:124. doi: 10.1186/s12889-023-15042-x (PMC9847128; doi:10.1186/s12889-023-15042-x)
Supplement: Supplementary file 1 — Additional file 1. A flow chart of the study participants included in the present study who were originally recruited from Songjiang District, Shanghai, China as part of the Shanghai Suburban Adult Cohort and Biobank (SSACB). * Inconsistent number is due to overlapped number of participants between data sources. [file 12889_2023_15042_MOESM1_ESM.pdf]

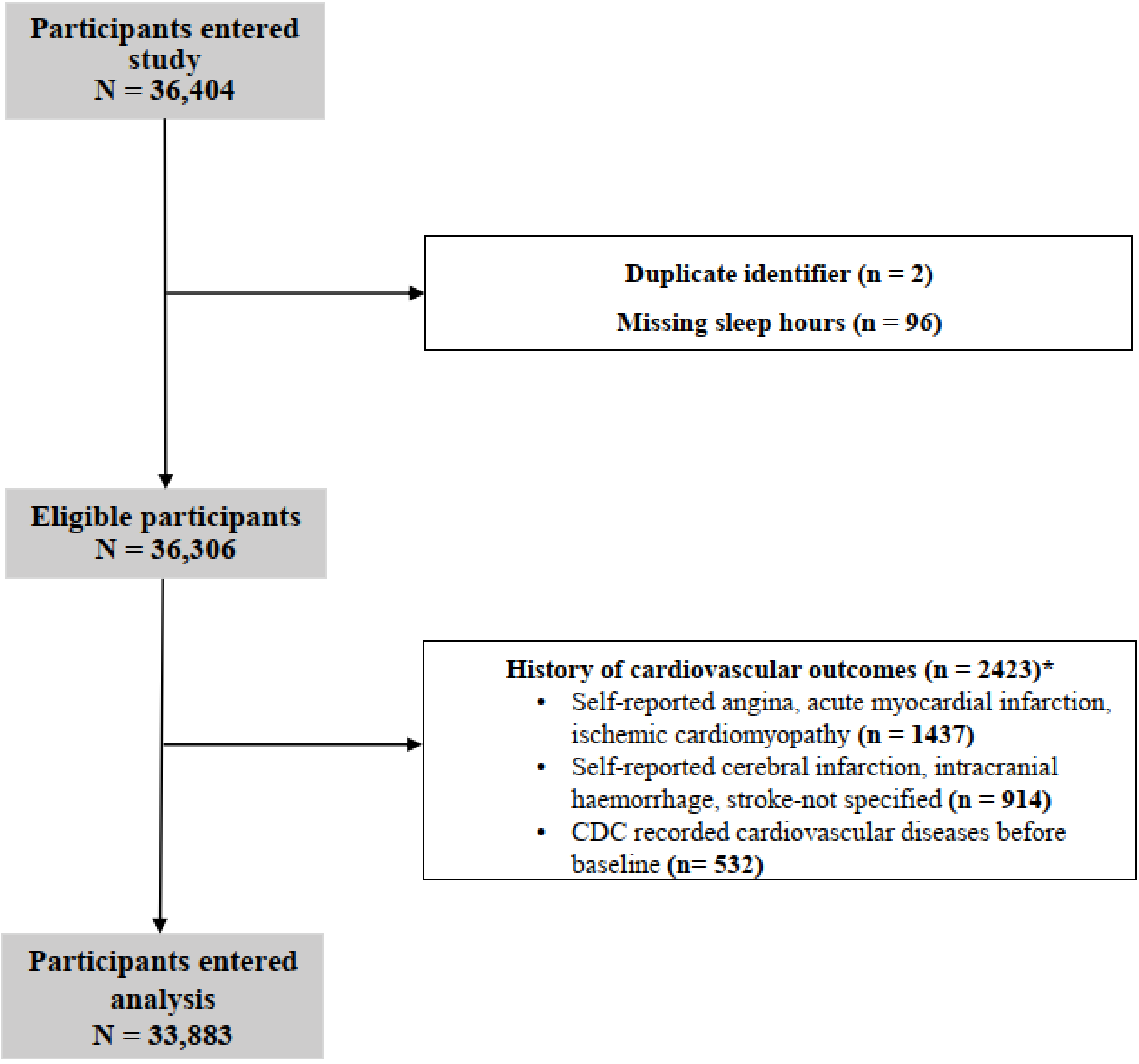

Additional file 1. A flow chart of the study participants included in the present study who were originally recruited from Songjiang District, Shanghai, China as part of the Shanghai Suburban Adult Cohort and Biobank (SSACB). * Inconsistent number is due to overlapped number of participants between data sources
