# Supplementary material for: Relationship of sleep duration with incident cardiovascular outcomes: a prospective study of 33,883 adults in a general population
Source: BMC Public Health. 2023 Jan 18;23:124. doi: 10.1186/s12889-023-15042-x (PMC9847128; doi:10.1186/s12889-023-15042-x)
Supplement: Supplementary file 4 — Additional file 4. Cumulative incidence curve in all participants (a) and participants ≥50y (b). Sleephg: sleep hour group. 1: "≤7 h"; 2: ">7 h to ≤ 8h"; 3: ">8 h to ≤9 h"; 4: ">9". CIF = cumulative incidence function. [file 12889_2023_15042_MOESM4_ESM.pdf]

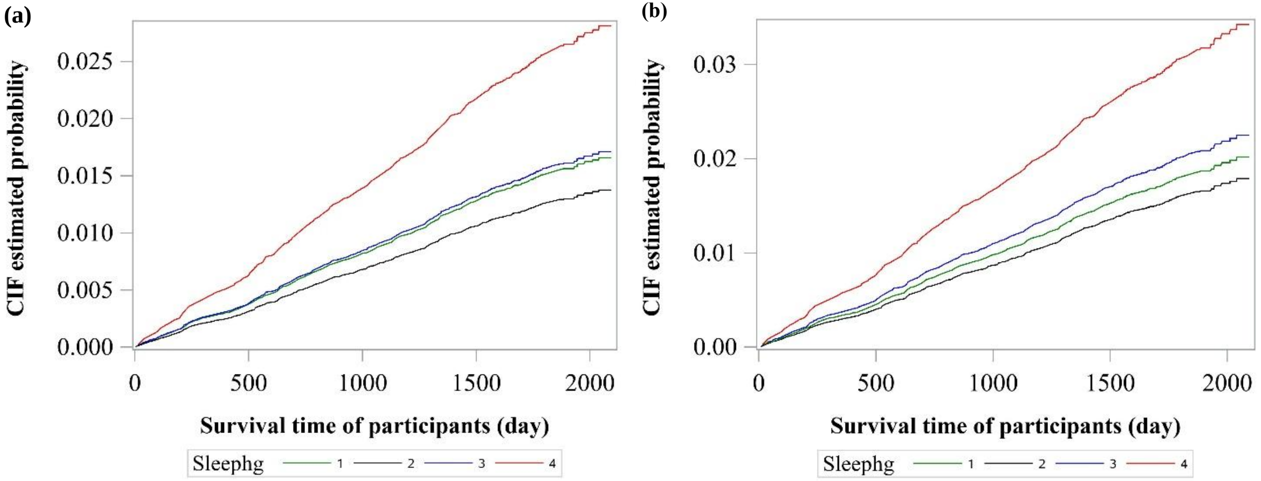


**Additional file 4.** Cumulative incidence curve in all participants (a) and participants ≥50y (b). Sleephg: sleep hour group. 1: "≤7 h"; 2: ">7 h to ≤8 h"; 3: ">8 h to ≤9 h"; 4: ">9 h". CIF = cumulative incidence function.
