# Supplementary figures and images for: Relationship of sleep duration with incident cardiovascular outcomes: a prospective study of 33,883 adults in a general population
Source: BMC Public Health. 2023 Jan 18;23:124. doi: 10.1186/s12889-023-15042-x (PMC9847128; doi:10.1186/s12889-023-15042-x)

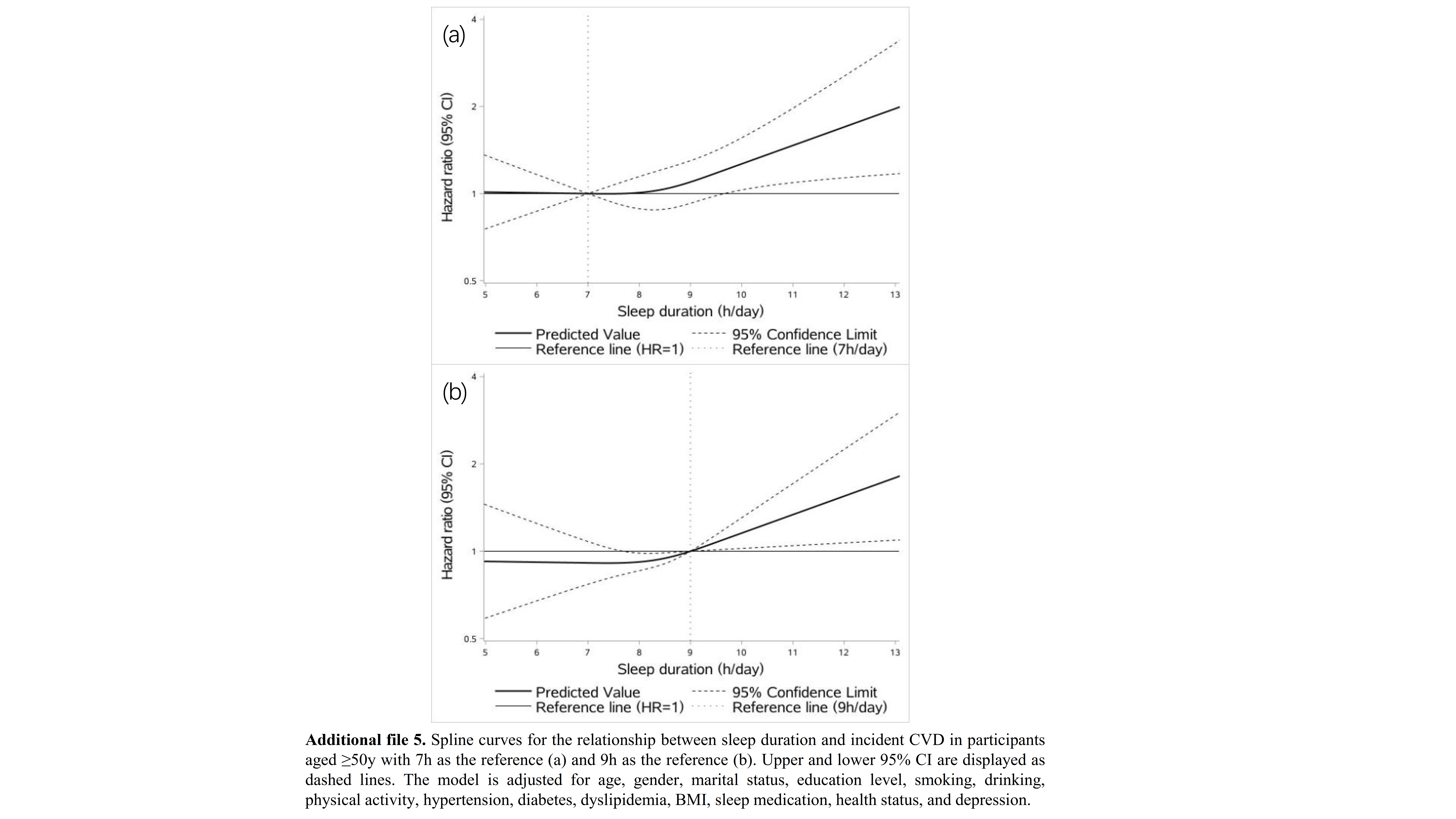

Supplement: Supplementary file 5 — Additional file 5. Spine curves for the relationship between sleep duration and incident CVD in participants aged ≥50y with 7h as the reference (a) and 9h as the reference (b). Upper and lower 95% CI are displayed as dashed lines. The model is adjusted for age, gender, martial status, education level, smoking, drinking, physical activity, hypertension, diabetes, dyslipidemia, BMI, sleep medication, health status, and depression. [file 12889_2023_15042_MOESM5_ESM.png]
